# Editorial: Biocentric development: studies on the consequences of COVID-19 towards human growth and sustainability

**DOI:** 10.3389/fpsyg.2023.1176314

**Published:** 2023-07-20

**Authors:** Marcus Stueck, Dian Veronika Sakti Kaloeti, Hamidrezah Kankeh, Mehrdad Farrokhi, Mariola Bidzan

**Affiliations:** ^1^International Biocentric Research Academy (IBRA), Leipzig, Germany; ^2^Faculty of Psychology, Universitas Diponegoro, Semarang, Indonesia; ^3^Health in Emergency and Disaster Research Center, Social Health Research Institute, University of Social Welfare and Rehabilitation Sciences, Tehran, Iran; ^4^Division of Clinical and Health Psychology, Institute of Psychology, University of Gdansk, Gdańsk, Poland

**Keywords:** biocentric development, COVID-19, sustainability, human growth, Biodanza, Relative Biocentric Health Theory, spirituality and health, biocentric borders

This Research Topic on “*Biocentric development and COVID-19*” has two main focuses. Firstly, to publish studies that scientifically elaborate the positive and negative consequences of the COVID-19 situation and, secondly, to make deductions related to biocentric development as an alternative mission search. The focus is not primarily on overcoming the manufactured COVID-19 crisis from an anthropocentric perspective (anthro = human, center = center) but also from a biocentric approach. Why? The global COVID-19 events, climate catastrophes, and countless environmental catastrophes show a pattern, namely, that humans have increasingly detached themselves from nature and its totality through their way of life and inner attitudes. For example, through inner perspectives

- that nature and life are exploitable resources,
- that there is a material world that will grow indefinitely,
- that man is the reference point for all developments, and that all other life forms are subordinate to him.

If one forms the dual expression of these attitudes (dualization) to restore balance, at least mentally, three important biocentric statements emerge:

First, nature and its associated life have an intrinsic value that humans must experience effectively. Toro (2010) described the importance of the inner experience in education, therapy, and personal growth and how to develop it using the biocentric method of “Biodanza.” This method, aiming to increase reverence for life is well-researched (Stueck and Tofts, 2016; Stueck and Villegas, 2018; Stueck et al., 2019).

Second, there is a material and a non-material, spiritual level that needs to be integrated with life and health and which is not a matter of belief but of logical deduction and increasing research (MacDonald et al., 2015; Stueck, 2021; Dewi et al., 2023).

Thirdly, that man is not only the anthropocentric and egocentric reference point of life but there is also a biocentric reference level (bios = life, center = center). This means that there is a connection between outer and inner natural space, as Stueck (2021) stated based on Naess (1989, 1998). In the “Deep Ecology”

approach they postulated that nature, the “complex of living beings,” is unfortunately seen as something separate from humans. Biocentric methods (interventions) to experience an observe the inner natural space are among others, Biodanza and Meditation (see Figure 1, point 9).

**Figure 1:**
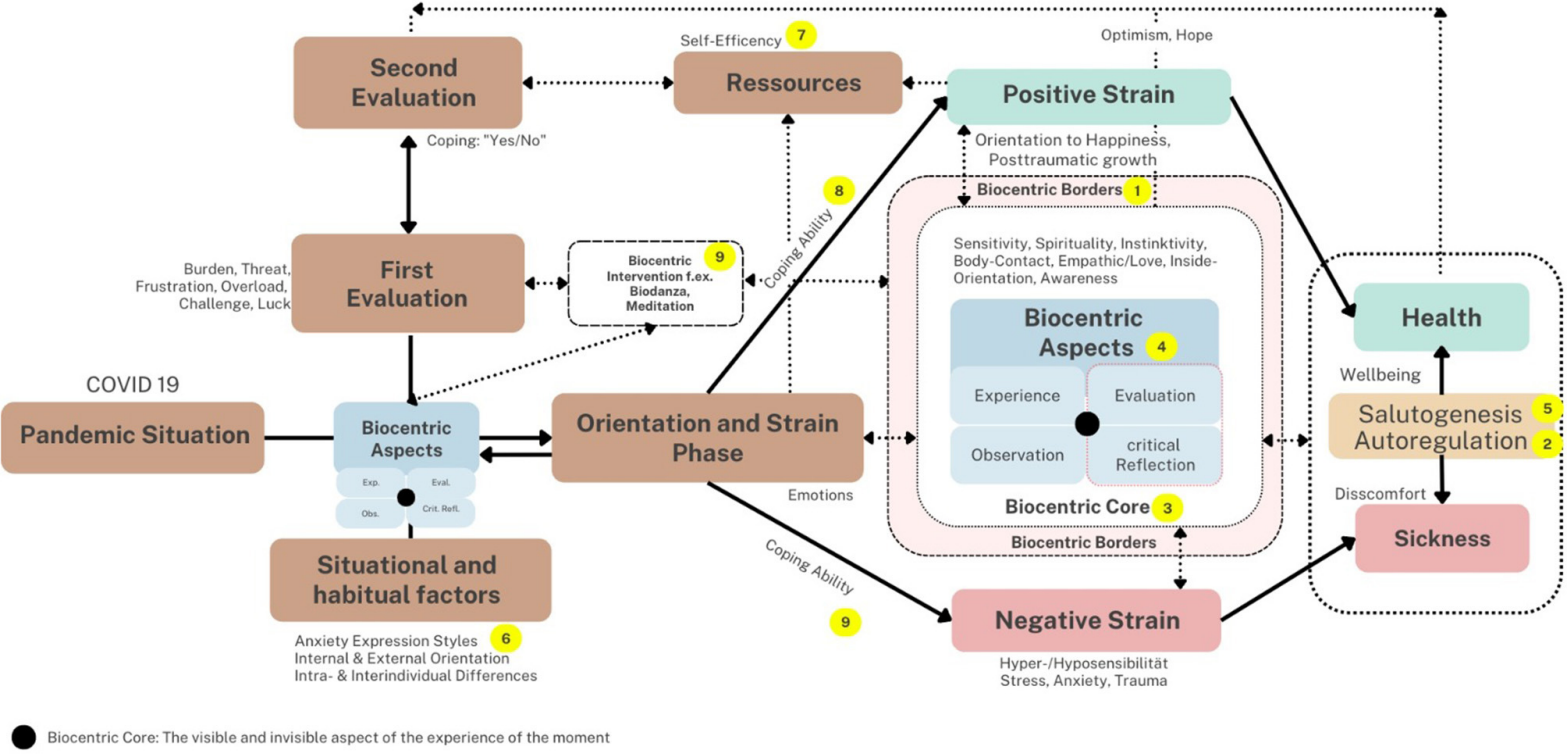
The Relative Biocentric Health Theory related to pandemic situations (Stueck, 2021, take-out - Figure 2).

What is the biocentric reference level? Schweitzer (1966) already described it as a way of life where individuals live with compassion and respect for all living things—humans, animals, and plants. Toro (2010) developed ideas on a biocentric principle and education, which emphasise about the protection of life and thus the affective connection (empathy) to oneself, others, and all life forms in nature. This includes expanding ethical awareness about the intrinsic value of life and nature at the center of consideration and research. Stueck (2021) defined and studied it in a Relative Biocentric Health Theory (RBHT) related to COVID-19, so called biocentric borders (see Figure 1, number 1), which are external and internal factors that hinder the normal process of autoregulation (see Figure 1, number 2) in the biocentric core (see Figure 1, number 3) of a living system. In humans, this perspective decreases effective communication and empathy for all natural life forms and the empathy for oneself (see Figure 1, number 4) and salutogenesis (see Figure 1, number 5). Research has shown that biocentric borders, like chronic stress, exhaustion, and hypersensitivity, lower the humans' empathic behavior (Stueck, 2008; Stueck et al., 2013). This “dehumanization” because of the biocentric borders (see Figure 1, number 1) of chronic stress and exhaustion, combined with a missing empathy, are accompanied by other affective pathologies. This includes addictions (Kaloeti and Kusnadi, 2022), but also the devaluation of others (racism, discrimination, bullying) (Kaloeti et al., 2021), fears of nature, fears of life, fears to be empathic and an inability to express oneself or to communicate (Toro, 2010). For this reason, further research on a biocentric evidence-based intervention, e.g. “School of Empathy,” for children and adults would be helpful (Stueck, 2010; Stück, 2013; Widiasmara et al., 2018).

Related to this model (see Figure 1), the topic articles in this Research Topic on “*Biocentric development and COVID-19*” can be categorized into three aspects:

The first aspect is research on Biocentric Borders during COVID-19 (Figure 1, point 1). Biocentric borders can hinder the ethics of coexistence and the deeper connection of human beings with themselves, others, and with nature. In this context, the article by Grabowski et al. found that the associated stress levels during the lockdown in Italy and Poland were related to higher activity levels. Furthermore, it was reported that less isolation correlated with less stress. The importance and the effects of social connection and support in combination with functioning emotional coping strategies on reducing stress, anxiety, and depression were underlined in a second article by Akbar and Aisyawati on this topic.

The second aspect explored by articles in this Research Topic is research about the balance between the internal and external orientation (see Figure 1, point 6) of people's perceptions during COVID-19. In this respect, the article by Nomura et al. is a significant contribution to the importance of counseling to increase reflexive activity (internal orientation) in students in difficult mental situations during COVID-19 to prevent depression and suicide. The better people can reflect on internal states, self-defense mechanisms, and dysregulated non-biocentric attitudes, the healthier they become or remain during COVID-19 (see Figure 1, point 2, 5, 6).

In studies on Relative Biocentric Health Theory (RBHT, Stueck, 2021) during COVID-19, people who developed a higher inner orientation during the pandemic crisis, e.g., by practicing psychotherapy, Biodanza, or meditation, were more connected to their “biocentric core” (Stueck, 2021). This means they are significantly more peaceful, autonomous, empathic, and capable of love (see Figure 1, point 3, 4). They also show different interpretations of the pandemic situations and less problematic anxiety expression styles, such as fewer sensitizers and displacement patterns (Mueller-Haugk and Stueck, 2023, see Figure 1, point 6). Unfortunately, during the COVID-19 lockdowns, no government-sponsored programs that systematically promoted inner orientation in children and adults and addressed people's inner problems even though scientists suggested the use of reflexive methods to increase self-efficacy in hospital staff (Bidzan et al., 2020, see Figure 1, resources, point 7) or self-management and psychological strategies to overcome difficulties during COVID-19 (Khankeh et al., 2021, 2022, see Figure 1, point 8).

The final aspect explored in this Research Topic includes research on inter- and intra- individual differences during COVID-19: this third category of articles on “*Biocentric development and COVID-19*” concerns people's inter- and intra- individual differences, which should be regarded when intervention strategies are selected because every human being is unique.

Unfortunately, this ability to differentiate has been missing from pandemic management in many countries (Khankeh et al., 2020; Bidzan-Bluma et al., 2021) (see Figure 1, point 6). The third biocentric basic assumption is explored in the topic through three articles. The first article by Candeias et al. concludes that the quality of life, optimism, and wellbeing are affected differently during the pandemic. This depends on the country and age group, suggesting individual differences between cultures and age groups and the need for specific interventions. A second article on this topic, by Islam et al. investigates the particular aspect of coping with COVID-19 and examines public health initiatives in Bangladesh that use biocentric approaches to mitigate the pandemic's potential financial and psychological impact on impoverished urban dwellers in Bangladesh. A third topic article in the context of interindividual differences during COVID-19 by Kaloeti et al. takes up gender differentiation concerning COVID-19, finding that women in Indonesia were more vulnerable to traumatic reactions.

The seven articles included in this topic indicate that there is a need for further research on these three biocentric aspects and that scientific studies on biocentric fields of action in combination with anthropocentric methods need to be conducted. This will ensure a practical transfer of the biocentric ideas in different working areas, e.g., to treat Long-COVID illnesses or to strengthen biocentric resources and enable humans for biocentric growth and sustainable development.

## Author contributions

DK finalized the manuscript. All authors contributed to the conception and design of the editorial, manuscript revision, read, and approved the submitted version.

## References

[B1] Bidzan M. Bidzan-Bluma I. Szulman-Wardei A. Stueck M. Bidzan M. (2020). Does self- efficacy and emotional control protect hospital staff from covid-19 anxiety and ptsd symptoms? Psychological functioning of hospital staff after the announcement of covid-19 coronavirus pandemic. Front. Psychol. 23, 552583. 10.3389/fpsyg.2020.552583 33424673 PMC7785971

[B2] Bidzan-Bluma I. Bidzan M. Jurak P. Bidzan L Knietzsch J Stueck M. Bidzan M. (2021). Polish and German population study of quality of life, well-being, and life satisfaction in older adults during the covid-19 pandemic. Front. Psychiatry 11, 585813. 10.3389/fpsyt.2020.585813 33281646 PMC7705096

[B3] Dewi A. K. Zulaifah E. Sari Utami D. Stueck M. (2023). Islamic Psychology–A Integrative Dialoque. New York, NY: Peter Lang.

[B4] Kaloeti D. V. S. Kusnadi A. P. (2022). Related factors of internet addiction on adolescents during COVID-19 pandemic: systematic literature review. J. Educ. Health Commun. Psychol. 4. 10.12928/jehcp.v11i4.2467436061296

[B5] Kaloeti D. V. S. Manalu R. Kristiana I. F. Bidzan M. (2021). The role of social media use in peer bullying victimatization and onset of anxiety among Indonesian elementaryschool children. Front. Psychol. 12, 635725. 10.3389/fpsyg.2021.635725 33995192 PMC8113408

[B6] Khankeh H. Porebrahimi M. Karibozorg M. Farahani M. Ranjbar M. Ghods M.. (2022). Baseline and postintervention assessment of sexual violence and condom use among female sex workers in a semiurban African community. Soc. Health Behav. 5, 154–161. 10.4103/SHB.SHB_29_20

[B7] Khankeh H. R. Farrokhi M. Khanjani M. S. Momtaz Y. A. Forouzan A. S. Norouzi M.. (2021). The barriers, challenges, and strategies of COVID-19 (SARS-CoV-2) vaccine acceptance: a concurrent mixed-method study in Tehran City, Iran. Vaccines 9, 1248. 10.3390/vaccines9111248 34835179 PMC8620861

[B8] Khankeh H. R. Khorasani-Zavareh D. Roudini J. Pourvakhshoori N. Ahmadi S. Abbasabadi-Arab M.. (2020). Challenges, strategies, and the lessons learned from covid-19 to manage second wave: a qualitative multi-method study in the context of Iran. BMC Public Health 21, 1919. 10.1186/s12889-021-11973-5 34686165 PMC8532398

[B9] MacDonald D. A. Friedman H. L. Brewczynski J. Holland D. Salagame K. K. Mohan K.. (2015). Spirituality as a scientific construct: testing its universality across cultures and languages. PLoS ONE 10, e0117701. 10.1371/journal.pone.0117701 25734921 PMC4348483

[B10] Mueller-Haugk S. Stueck M. (2023). Relationship of anxiety management types and health- related variables of people during lockdown in a German sample. J. Health Emerg. Disast. Q. 8, 65–76. 10.32598/hdq.8.1.1

[B11] Naess A. (1989). Ecology, Community and Lifestyle. Cambridge: Cambridge University Press.

[B12] Naess A. (1998). Life's Philosophy: Reason and Feeling in a Deeper World. Athens: The University of Georgia Press.

[B13] Schweitzer A. (1966). The Teaching of Reverence for Life. London: Peter Owen Limited.

[B14] Stück M. (2013). “School of empathy: Introduction and first results,” in Contributions to Educational and Rehabilitation Psychology. Historical and Cross-Cultural Aspects of Psychology, ed E. Witruk (Frankfurt am M.: Peter Lang), 245–265.

[B15] Stueck M. (2008). “New ways: Yoga and Biodanza in the stress reduction for teacher,” in New Paths in Education and Psychology (Bd.1. Strasburg: Schibri).

[B16] Stueck M. (2010). Children, Researchers, Educators - Early Education in the Test. Contributions to Educational Health. Bd. 14. Strasburg: Schibri-Verlag.

[B17] Stueck M. (2021). The pandemic management theory: COVID-19 and biocentric development. Health Psychol. Rep. 9, 101–128. 10.5114/hpr.2021.103123 PMC10687539 38084288

[B18] Stueck M. Kaloeti D. V. S. Villegas A. Utami D. S. (2019). The influence of biodanza and school of empathy verbal - respectful communication on the ability to express emotions and needs: a pilot study among adults in Indonesia. Health Psychol. Rep. 7, 334–340. 10.5114/hpr.2019.88665

[B19] Stueck M. Schoppe S. Lahn F. Toro R. (2013). “What good is it to be empathise with someone without acting?,” in Investigation about the Integration of Prosocial Action into the Western Concept of Empathy in the Form of a Measurement Instrument for the Holistic Assessment of Empathy (ErgoMed/Practical Working Medicine), 38–46. Available online at: http://www.bildungsgesundheit.de/Publikationen/empa_6_2023_empathie_stueck-5.pdf

[B20] Stueck M. Tofts P. S. (2016). Biodanza effects on stress reduction and well-being: a review of study quality and outcome. J. Pedag. Psychol. Signum Temp. 8, 57–66. 10.1515/sigtem-2016-0018

[B21] Stueck M. Villegas A. (2018). Dance Towards Health. Strasburg: Schibri.

[B22] Toro R. (2010). Das system Biodanza. Offenbach: Tinto-Verlag.

[B23] Widiasmara N. Novitasari R. Trimulyaningsih N. Stueck M. (2018). School of empathy for enhancing children's well-being. Int. J. Soc. Sci. Human. 8, 230–234. 10.18178/ijssh.2018.V8.966

